# The Disruption of Liver Metabolic Circadian Rhythms by a Cafeteria Diet Is Sex-Dependent in Fischer 344 Rats

**DOI:** 10.3390/nu12041085

**Published:** 2020-04-14

**Authors:** Héctor Palacios-Jordan, Miguel Z. Martín-González, Manuel Suárez, Gerard Aragonès, Begoña Muguerza, Miguel A. Rodríguez, Cinta Bladé

**Affiliations:** 1 Nutrigenomics Research Group, Department of Biochemistry and Biotechnology, Universitat Rovira i Virgili, 43007 Tarragona, Spain; hector.palacios@eurecat.org (H.P.-J.); miguelmg.1991@gmail.com (M.Z.M.-G.); manuel.suarez@urv.cat (M.S.); gerard.aragones@urv.cat (G.A.); begona.muguerza@urv.cat (B.M.); mariacinta.blade@urv.cat (C.B.); 2 Eurecat, Centre Tecnològic de Catalunya, Centre for Omic Sciences (COS), Joint Unit Univeristat Rovira i Virgili-EURECAT, Unique Scientific and Technical Infrastructures (ICTS), 43204 Reus, Spain

**Keywords:** circadian rhythms, obesity, gender, liver, metabolomics

## Abstract

Circadian rhythms are ~24 h fluctuations of different biological processes that are regulated by the circadian clock system. They exert a major influence on most of the metabolism, such as the hepatic metabolism. This rhythmicity can be disrupted by obesogenic diets, fact that is considered to be a risk factor for the development of metabolic diseases. Nevertheless, obesogenic diets do not affect both genders in the same manner. We hypothesized that the circadian rhythms disruption of the hepatic metabolism, caused by obesogenic diets, is gender-dependent. Male and female Fischer 344 rats were fed either a standard diet or a cafeteria diet and sacrificed at two different moments, at zeitgeber 3 and 15. Only female rats maintained the circadian variations of the hepatic metabolism under a cafeteria diet. Most of those metabolites were related with the very low density lipoprotein (VLDL) synthesis, such as choline, betaine or phosphatidylcholine. Most of these metabolites were found to be increased at the beginning of the dark period. On the other hand, male animals did not show these time differences. These findings suggest that females might be more protected against the circadian disruption of the hepatic metabolism caused by a cafeteria diet through the increase of the VLDL synthesis at the beginning of the feeding time.

## 1. Introduction

Almost all mammalian cell types express clock genes and are synchronized by the circadian clock system, which is located in the suprachiasmatic nucleus (SCN) of the hypothalamus and ensures that internal physiology is synchronized with the external environment [1]. Most of the hepatic metabolisms present circadian rhythms [2]. In this sense, glucose and lipid metabolism is subjected to a time circadian control [3,4,5]. Glucagon and insulin, synthesized in and released from pancreatic α and *β* cells, regulate these pathways, and daily plasma rhythms in these hormones have been identified in rodents and humans [6]. Moreover, cholesterol is also synthesized in a circadian manner in the liver through the circadian expression of β-hydroxy-3-methylglutaryl-CoA reductase (HMG-CoA) [7]. Additionally, bile acids are synthesized from cholesterol exclusively in the liver by the rate-limiting enzyme cholesterol 7α-hydroxylase (CYP7A1), and it exhibits a well-documented rhythm of mRNA expression in rodents and an enzyme activity in human serum [8,9]. Moreover, enzymes that regulate fatty acid synthesis, such as the elongation of very long chain fatty acids protein 3 (ELOVL3), ELOVL6, and fatty acid synthase (FAS), present rhythmic expression patterns [10]. Furthermore, core clock and clock-controlled genes have additional roles in glucose metabolism in the liver. In this regard, Zhang et al. [11] showed that the overexpression of cryptochrome 1 (*Cry1*) decreases blood glucose levels and increases insulin sensitivity in diabetic mice. Altogether, these findings suggest that metabolomics is a useful tool to quantify 24-h oscillations of the hepatic metabolites, and consequently, the hepatic internal time [12].

The master circadian clock is disturbed in diet-induced obesity, and circadian misalignment has been identified as a risk factor for developing metabolic disorders [13,14]. Under an obesity status, clock gene expressions are disrupted within the hypothalamus, the liver, and adipose tissue, as well as the rhythmicity of hormones and nuclear hormone receptors involved in fuel utilization, such leptin and thyroid stimulating hormone (TSH), and testosterone in mice, rats and humans [15,16,17]. Particularly, the disruption of hepatic circadian rhythms might lead to non-alcoholic liver diseases (NAFLD) [18]. Kohsaka et al. [15] determined that the amplitude of the hepatic BMAL1 expression was reduced during the light period, whereas the PER2 expression was decreased during the dark period in mice fed a high fat diet.

Notably, obesogenic diets do not act in the same manner between both genders [19]. Van Nas et al. [20] observed a strong correlation between body fat and lipids, and a sex-specific group of genes in adipose tissue and liver. In addition, a study performed by Carmona-Alcocer et al. [21] demonstrated that plasma parameters associated with metabolic syndrome, such as leptin and insulin, are modulated in a sex-dependent manner by obesogenic diets. Circadian rhythms might participate in this differential behavior between genders. Supporting this idea, Kruppel-like factor 10 (KLF10), which is a transcription factor regulated by CLOCK and BMAL, regulates the expression of genes involved in glycolysis and gluconeogenesis [22]. Interestingly, the loss of KLF10 affects glucose metabolism in a sex-specific manner, inducing postprandial and fasting hyperglycaemia in male mice, whereas female mice remained normoglycemic [23]. Additionally, Perez-Mendoza et al. [24] found more altered the hepatic BMAL1 expression in female mice fed a high-fat diet than in male ones which regulates PPARs, strongly related with the lipid metabolism.

## 2. Materials and Methods

### 2.1. Animal Experimental Procedure

The investigation was carried out in accordance with the ethical standards and the Declaration of Helsinki and was approved by the Ethics Review Committee for Animal Experimentation of the Universitat Rovira I Virgili (reference number 9495 by Generalitat de Catalunya).

A total of 64 eight-week-old female and male Fisher 344 rats, 8 rats per group (*n* = 8), were purchased from Charles River Laboratories (Barcelona, Spain). Female and male rats were housed separated in animal quarters at 22 °C with a light/dark period of 12 h (light from 08:00 to 20:00 h). The animals were randomly divided into eight groups depending on the diet, gender and the sacrifice hour: 11:00 (ZT3) or 23:00 (ZT15). There were 4 control groups, one group for each time for both genders. The control groups (STD) were fed a standard chow diet (STD Panlab A04, Panlab, Barcelona, Spain) and water ad libitum. A cafeteria diet consisting of biscuit with pate, biscuit with cheese, bacon, carrots and sweetened milk (20% sucrose *w/v*) in addition to the standard chow diet was used as an obesogenic diet in the obese groups (CAF). The STD diet chow diet had a caloric breakdown of 14% protein, 8% fat, 73% carbohydrates, and 0.3% of NA, whereas the calorie breakdown of the CAF diet was: approximately 14% of proteins; 35% of fat and 51% of carbohydrates. The CAF diet administration was maintained to the end of the experiment. After nine weeks, the rats were fasted for 3 h after ZT0 or ZT12 and then sacrificed by decapitation. Blood from the saphenous vein was collected, and serum was obtained by centrifugation (1500× *g*, 20 min, 4 °C). The liver was harvested and immediately frozen in liquid nitrogen. Both serum and liver were stored at −80 °C until further use.

### 2.2. Biometric Parameters and Serum Analysis

Lean and fat measurements (in grams and percentage of body weight) were performed 1 week before the sacrifice using an EchoMRI-700^TM^ device (Echo Medical Systems, L.L.C., Houston, TX, USA). Body weight was monitored weekly until the end of the experiment. Body weight gain was calculated by subtracting the initial body weight from the final one. Food intake was determined weekly weighting the food used as cafeteria diet, and the caloric intake was calculated according to the caloric content of each food provide by the manufacturer. These variables were evaluated as physiological indicator of the obesity grade in the experimental animals (Supplementary Table S1).

Triglycerides (Triglycerides liquid (GPO method), QCA, Barcelona, Spain) in serum were analyzed by an enzymatic colorimetric assay kit following the instruction provided by the manufacturer. 

### 2.3. Liver Extraction Procedure for ^1^H NMR-Based Metabolomics Assays

Liver extraction was performed following the procedure described by Vinaixa et al. with slight modifications [25]. A portion of hepatic tissue (50 mg) was removed, flash-frozen, and manually homogenized using a micropestle in 1 mL of H_2_O/CH_3_CN (1/1). The homogenates were centrifuged at 15,000× *g* for 30 min at 4 °C. Supernatants (hydrophilic metabolites) were separated from the pellet (lipophilic metabolites) and lyophilized overnight to remove water for better NMR performance and, once dried, stored at −80 °C until further analysis. The lipophilic pellet extracts were subsequently extracted with 1 mL of a solution of CHCl_3_/CH_3_OH (2:1) and then vortexed, homogenized for 20 min, and centrifuged for 15 min at 6000× *g* at room temperature. The new lipophilic supernatant was separated and dried under N_2_ stream.

### 2.4. NMR Analyses

For NMR measurements, the hydrophilic extracts were reconstituted in 600 μL of D_2_O phosphate buffer (PBS 0.05 mM, pH 7.4, 99.5% D_2_O) which contained 0.73 mM trisilylpropionic acid (TSP). The dried lipophilic extracts were reconstituted with a solution of CDCl_3_/CD_3_OD (2:1) containing 1.18 mM tetramethylsilane (TMS). Both extracts were transferred into 5-mm NMR glass tubes for NMR measurement. The ^1^H NMR spectra were recorded at 300 K on an Avance III 600 spectrometer (Bruker, Germany) operating at a proton frequency of 600.20 MHz using a 5 mm PABBO broadband gradient probe. For aqueous extracts, one-dimensional ^1^H pulse experiments were carried out using the nuclear Overhauser effect spectroscopy (NOESY) presaturation sequence (RD–90°–t1–90°–tm–90° ACQ) to suppress the residual water peak, and the mixing time was set at 100 ms. Solvent presaturation with an irradiation power of 75 Hz was applied during recycling delay (RD = 5 s) and mixing time. The 90° pulse length was calibrated for each sample and varied from 9.95 to 10.06 µs. The spectral width was 12 kHz (20 ppm), and a total of 256 transients were collected into 64 k data points for each 1H spectrum. In the case of lipophilic extracts, a 90° pulse with a presaturation sequence (*zgpr*) was used to suppress the residual water signal of methanol. An RD of 5.0 s with an acquisition time of 2.94 s was used. The 90° pulse length was calibrated for each sample and varied from 9.92 to 10.04 µs. After 4 dummy scans, a total of 128 scans were collected into 64K data points with a spectral width of 18.6 ppm.

The exponential line broadening applied before Fourier transformation was 0.3 Hz. The frequency domain spectra were phased, baseline-corrected, and referenced to trimethylsilyl propinate or TMS signal (δ = 0 ppm) using TopSpin software (version 2.1, Bruker).

### 2.5. NMR Data Analysis

The acquired ^1^H NMR spectra were compared to references of pure compounds from the metabolic profiling AMIX spectra database (Bruker), HMDB, Chenomx NMR suite 8.4 software (Chenomx Inc., Edmonton, AN, Canada) and databases for metabolite identification. In addition, we assigned metabolites by ^1^H–^1^H homonuclear correlation (COSY and TOCSY) and ^1^H–^13^C heteronuclear (HSQC) 2D NMR experiments and by correlation with pure compounds run in-house. After pre-processing, specific ^1^H NMR regions identified in the spectra were integrated using the AMIX 3.9 software package.

### 2.6. Data Processing and Multivariate Analysis

Absolute concentrations derived from both lipophilic and hydrophilic extracts were arranged together in one single data matrix. Previously, data were scaled to unit variance to give all the identified metabolites the same weighing into the model. Data analysis and statistical calculation were performed with IBM Corp. (released 2013. IBM SPSS Statistics for Windows, Version 22.0. Armonk, NY, USA: IBM Corp). For the partial least square–discriminant analysis (PLS-DAs), MetaboAnalyst software 4.0 was used (https://www.metaboanalyst.ca/).

## 3. Results

### 3.1. Biometric Parameters Validated the Obesogenic Effect of the Diet

The biometric parameters confirmed that the cafeteria diet used in this experiment caused the obese status in the Fischer 344 rats. No significant differences on the initial body weight were found between the standard and cafeteria groups being the male rats more heavy than female ones. After 9 weeks of cafeteria diet feeding, the final body weight and body weight gain of the CAF groups were significantly higher than the standard ones. Moreover, the caloric intake was also increased in the group fed a cafeteria diet (Supplementary Table S1). 

### 3.2. Multivariate Chemometric Analysis of NMR Data

Due to the NMR sample limitations, only 57 samples were analyzed. The PCA methodology was used to detect putative outliers. PCA was performed using the whole spectra, removing the TSP or TMS, methanol, chloroform and water regions in both phases, aqueous and lipid. After alignment and normalization of the spectra, a total of 45 metabolites were found. In the aqueous phase, 28 metabolites were identified and integrated, and 17 metabolites in the lipid phase (Supplementary Tables S2–S5).

### 3.3. Multivariant Analyses Showed a Gender-Dependent Dysregulation of the Homeostatic Equilibrium of the Hepatic Metabolism Induced by a Cafeteria Diet

**Figure 1:**
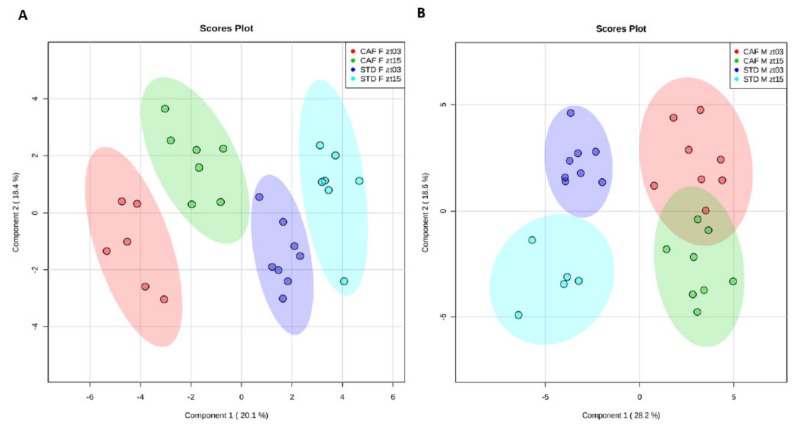
The 45 liver metabolites were used to set up a PLS-DA predictive model. It was done in both, females (**A**) and males (**B**). CAF F ZT3, cafeteria female group at Zeitgeber 3; CAF F ZT15, cafeteria female group at Zeitgeber 15; STD F ZT3, standard female group at Zeitbeger 3; STD F ZT15, standard female group at Zeitbeger 15; CAF M ZT3, cafeteria male group at Zeitgeber 3; CAF M ZT15, cafeteria male group at Zeitgeber 15; STD M ZT3, standard male group at Zeitbeger 3; STD M ZT15, standard male group at Zeitbeger 15.

First, we analyzed whether the sets of hepatic metabolites differed between both times, ZT3 and ZT15. To this end, supervised PLS-DA models were applied to determine how diets influenced the homeostatic equilibrium of the hepatic metabolites depending on gender. The PLS-DAs were performed by comparing both times and diets for females (Figure 1A) and males (Figure 1B) separately.

In females, clear grouping according to the diet was observed using the PLS-DA approach. Moreover, the control female rats showed a separation by ZT. Notably, ZT separation was also very evident in female rats fed a cafeteria diet. In the constructed PLS-DA model, the R^2^ and Q^2^ values were 0.990 and 0.942, respectively, employing the 4th principal component. To validate the model, permutation testing was performed allowing 1000 permutations. The significance of the model was *p* < 0.001, verifying the validity of the model. Therefore, although the metabolites that differ between both times in the livers of female rats fed control or cafeteria diet were different, female rats maintained their time clustering when fed a cafeteria diet.

In males, as well as in females, a clear clustering by diet was found. Furthermore, there was also grouping in the control male rats according to the ZT, as it did in females. Although, the lack of samples of the STD M ZT15 group could slightly modify the clustering observed in the standard male animals. However, in contrast to female rats fed a cafeteria diet, male rats fed a cafeteria diet did not present significant differences between both ZTs, as it did in standard-fed male animals. The R^2^ and Q^2^ values were 0.963 and 0.839, respectively, employing the 3rd principal component. The model significance was *p* < 0.001, which confirms the model validity. These results indicated that male rats were more susceptible to the disruption of the homeostatic equilibrium of the hepatic metabolism induced by an obesogenic diet than female rats.

Altogether, the separation between both ZTs in the PLS-DA robustly indicates that the hepatic metabolism exhibited rhythmicity in the animals fed a standard diet between ZT3 and ZT15 independently of the gender. However, only female rats were capable of maintaining circadian variations under an obesogenic diet.

### 3.4. The Differences between both Times are Gender-Dependent

Once we determined that the hepatic metabolism has circadian rhythmicity due to the differences observed in the homeostatic equilibrium, we further studied which specific metabolites showed differences between ZT3 and ZT15 and whether these characteristic metabolite changes were specific to gender and/or could be modified by an obesogenic diet (Supplementary Tables S2–S5).

In control rats, the metabolites shared in both genders that were found to be higher at ZT3 were fumarate and ascorbate, while choline and lysine were increased at ZT15. In males, inosine and alanine had higher concentrations at ZT3, and 3-hydroxybutyrate (3-OHB), creatinine and sphingomyelin were increased at ZT15. In contrast, females presented lower levels of isoleucine, omega-3, and docosahexaenoic acid (DHA) at ZT3. Therefore, some metabolites that differed between both times were specific for each gender in normal and healthy rats.

Rats fed a cafeteria diet strongly modified the metabolites that differed between both time in the control ones. Notably, the metabolites shared by both genders in control rats were lost. However, 3-OHB, glutamine, free cholesterol, esterified cholesterol, phosphatidylethanolamine and the sum of arachidonic acid and eicosapentaenoic acid (ARA + EPA) showed differences between ZT3 and ZT15 in rats fed a cafeteria diet, presenting higher concentrations of these metabolites at ZT15. Additionally, the number of metabolites that differ between both times in females fed a cafeteria diet was higher than those that differ in control females. The female group exhibited differences in isoleucine, omega-3 and DHA, such as in control female rats, but also differences in fumarate, choline, betaine, phenylalanine, tyrosine, valine, TAG, DG, total phospholipids, phosphatidylserine, phosphatidylcholine, sphingomyelin, and plasmalogen, metabolites that showed higher concentrations at ZT15. In contrast, male rats fed a cafeteria diet showed a reduced number of metabolites that differ between both times. In this sense, only total cholesterol and creatine showed differences in their concentrations in obese male rats, with low levels at ZT3. These results indicated that the obesogenic diet used altered the metabolite rhythm characteristics of healthy rats. Moreover, as for normal rats, metabolic differences were specific for each gender. In addition, the male rats fed a cafeteria diet presented low levels of PC and choline at ZT15, which are indicative of steatosis [26].

**Figure 2:**
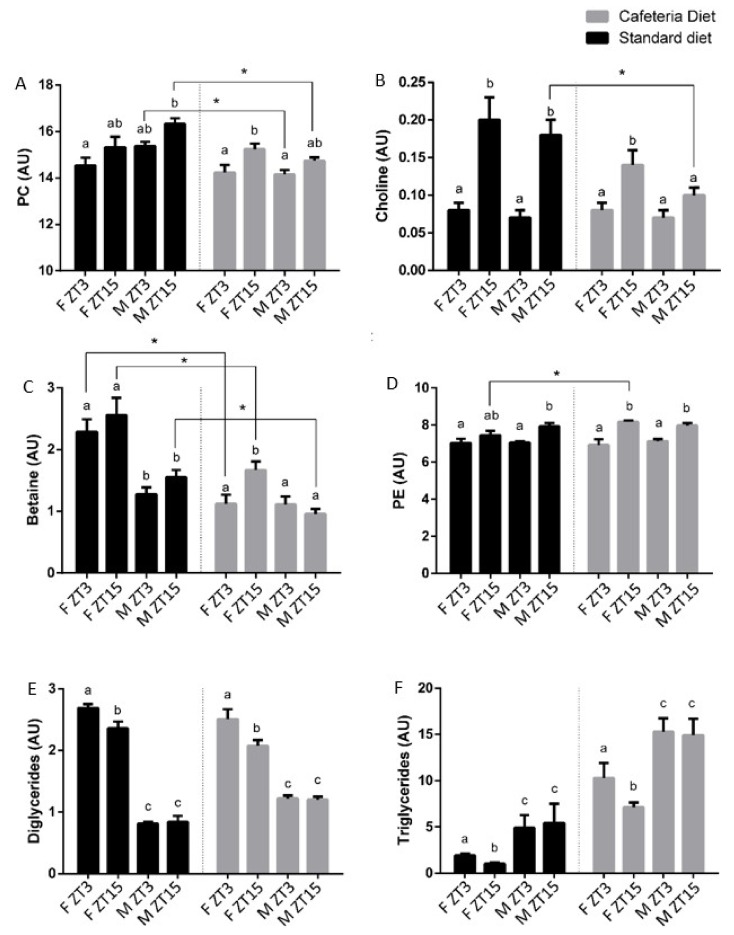
The concentration of the metabolites in the liver of female and male Fischer 344 rats fed with cafeteria or standard diet: phosphatidylcholine (**A**), choline (**B**), betaine (**C**), phosphatidylethanolamine (**D**), diglycerides (**E**), and triglycerides (**F**). Data are expressed as the mean ± SEM (*n* = 8). ^abc^ Mean values with unlike letters were significant different among groups (one-way ANOVA and Duncan’s *post hoc* test). * denotes a significant difference between groups (*p* < 0.05; t-Student). PC, phosphatidylcholine; PE, phospatidylethanolamine.

Since the PLS-DA exhibited a clustering by ZT only in female animals fed a cafeteria diet and the analysis of variance showed that the variation in time of metabolites was gender specific, we considered metabolites that differ in female obese rats as relevant metabolites maintaining circadian rhythms in the hepatic metabolism. These metabolites were 3-OHB, fumarate, choline, betaine, phenylalanine, valine, TAG, DG, sphingomyelin, plasmalogen, total phospholipids, PC, phosphatidylserine, omega-3, DHA and ARA+EPA. Notably, most of the metabolites are related to the metabolism of the VLDL packaging, indicating that VLDL synthesis and secretion in the liver is a key process to maintain the metabolite homeostasis under the cafeteria diet (Figure 2).

### 3.5. PC Changes in the Livers of Female Rats Fed a Cafeteria Diet were Negatively Correlated with Plasma TAG

VLDL secretion by the liver is directly related to plasma TAG levels. Thus, we further analysed plasma TAG levels in 26 CAF animals. There was no significant difference in plasma TGAs between ZT3 and ZT15 in any gender. The TAG concentrations in obese females were 205.72 ± 39.81 mg/dL and 156.65 ± 19.11 mg/dL at ZT3 and ZT15, respectively. In obese males, the values were 360.79 ± 25.37 mg/dL at ZT3 and 397.5 ± 23.52 mg/dL at ZT15.

**Figure 3:**
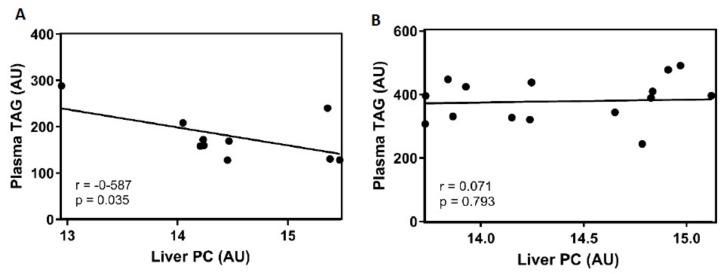
Plot of the significant associations between the live phosphatidylcholine and plasma triglycerides in female (**A**) and male (**B**). Each plot presents each Pearson r correlation value and the corresponding *p*-value. *p*-value < 0.05 were considered statistically significant.

PC in the liver is strongly related to VLDL packaging and, therefore, with TAG secretion [27]. To determine whether the differences between both time of hepatic PC are related to VLDL secretion, correlation analysis between PC in liver and plasma TAG was performed in both genders. Figure 3A exhibits a negative correlation between the PC levels in the liver and the plasma TAG concentration in CAF-fed female animals. These results suggest a biological role for the liver PC levels in the clearance of plasma TAG. Moreover, in Figure 3B, CAF-fed male rats did not show any correlation between both parameters.

## 4. Discussion

In the present study, we showed that the homeostatic equilibrium between two ZTs of the 24 h cycle, modulated by daily rhythms, were affected by an obesogenic diet in a different way in female and male rats. Multivariate analyses were carried out with 45 metabolites in both genders at ZT3 and ZT15. The results showed a clear clustering depending on the diet and the ZT in the female rats on both diets. However, the male rats exhibited a clear clustering depending only on the diet with a slight clustering between ZTs in control males that was lost in males fed a cafeteria diet. These results suggested that males, in contrast with the females, were not able to tolerate a cafeteria diet, thus exhibiting a loss of the homeostatic equilibrium due to a total or partial dysregulation of daily rhythms on the hepatic metabolism. During the day, CLOCK and BMAL1 heterodimerize to drive rhythmic expression of downstream target genes, which in turn regulate diverse metabolic processes and their homeostasis [28]. Unbalanced diets might cause disruptions on the internal clock system, which constitute risk factors for the metabolic syndrome disorders such as type 2 diabetes mellitus, cardiovascular diseases, thrombosis and inflammation [29]. In addition, the loss of daily variations has previously been observed in peripheral tissues in animals subjected to an obesogenic diet [30,31]. Previous studies have demonstrated that the chronic consumption of high-calorie diets disrupt the correct functionality of the mammalian circadian clock [15]. Whereas central clock genes are mainly synchronized by the light, peripheral clock genes are principally regulated by the diet and the feeding time [32].

As mentioned previously, our results indicated that female rats could maintain the differences between both ZT3 and ZT15 in the hepatic metabolism, presenting better metabolic flexibility under a cafeteria diet. The protective effect of estrogens against the disturbances caused by obesogenic diets might be one reason to explain why the obese female animals conserved the homeostatic equilibrium of the hepatic metabolites [33,34].

Most of the metabolites that showed differences between both genders and, specifically, those that helped to explain the capacity of the female rats fed a cafeteria diet to keep the homeostatic equilibrium of the hepatic metabolism, were both directly and/or indirectly related to VLDL packaging. The most abundant phospholipid in the VLDLs is PC, representing 70% of phospholipids [35]. PC is not only the most abundant phospholipid in VLDL but is also required for its assembly [27]. Hence, reduced levels of hepatic PC are associated with an impairment in VLDL secretion from the liver [36]. In control animals of both genders, there was no difference between the ZTs in PC. Otherwise, the obese female rats exhibited daily variations in PC with higher concentrations at ZT15, whereas the obese male rats did not show differences at all. Furthermore, the PC concentration in CAF-fed male rats was lower than that in STD-fed male rats. Since low levels of PC have been associated with fatty acid liver disease [37], our results suggest that obese male rats have a higher degree of steatosis-lipid accumulation than obese female animals, which can maintain the homeostatic rhythmicity of PC.

PC could be derived either from the phosphorylation of choline, through the cytidine diphosphate-choline pathway (CDP-Choline), or from the phosphatidylethanolamine N-methyltransferase pathway (PEMT), and several metabolites of these pathways showed rhythmicity in this study [38,39].

For the CDP-choline pathway, choline is the first metabolite of the pathway, which is converted to CDP-choline after a set of reactions, and it is esterified with diglycerides (DAG) by choline phosphotransferase to produce PC [38]. In control rats, the levels of choline in the liver were significantly higher at ZT15 than at ZT3 in both genders. Notably, this choline time differentiation was only maintained in the female animals fed a cafeteria diet, which had a higher concentration at the same time than in the control animals, i.e., ZT15. DAG, which are necessary to esterify CDP-choline, also exhibited daily variations in both diets in the female rats but presented lower levels at ZT15. The fact that females fed a cafeteria diet presented higher levels of choline and PC and lower levels of DAG at ZT15 than at ZT3 suggested that PC production is increased through the CDP-choline pathway at ZT15 in females.

In addition to the CDP-choline pathway, the PEMT pathway generates PC from PE through 3 transmethylation reactions using S-adenosyl methionine (SAM) as a methyl donor and is catalyzed by phosphatidylethanolamine N-methyltransferase (PEMT) [40]. Choline is oxidized to betaine, which acts as a methyl donor for the synthesis of methionine and dimethylglycine from homocysteine [41,42]. Finally, the enzyme methionine adenosyltransferase transforms methionine into SAM [43]. In this study, betaine presented variations only in female rats fed a cafeteria diet, with the highest concentration at ZT15. Additionally, PE levels in the liver differ between ZT3 and ZT15 control males and in both genders fed a cafeteria diet. In all these cases, PE concentration was higher at ZT15 than at ZT3. Notably, female rats fed a cafeteria diet presented differences in choline, betaine and PE levels in the liver between both times, with higher levels at the same time, at ZT15. Thus, females fed a cafeteria diet also showed clear rhythmicity in the metabolites related to the PEMT pathway, while the rest of the groups presented very few or no daily variations on the metabolites related to this pathway. This fact strongly suggests that females fed a cafeteria diet increased the production of PC at ZT15 through the activation of both choline pathways at this ZT, as revealed by the increase in several metabolites related to both the methyl donor and the PEMT pathways. This finding indicates that PC might be not only increased at ZT15 by the CDP-choline pathway but also could be by the PEMT pathway in female animals fed a cafeteria diet.

In contrast to females, the choline levels at ZT15 in the liver of males fed a cafeteria diet were lower than in the control ones, indicating a low choline disposal at this ZT. Choline deficiency has been reported to be related to decreased methylation capacity, perturbed phosphatidylcholine synthesis, impaired VLDL secretion, decreased PPARα signaling, and altered lipid metabolism, influencing the progression from a healthy liver to fatty liver [26]. Therefore, this choline scarcity in the liver could be relevant for the fragility shown by male rats to resist an obesogenic diet.

The VLDLs are the primary vehicle for the transport of TAG [44]. TAG may be assembled from FAs derived from plasma NEFAs, chylomicron remnants, or from *de novo* lipogenesis [45]. Once the VLDLs are assembled with the TAG, they are secreted to the blood [46,47]. Hence, the ability to keep or generate daily variation in the metabolites related to the packaging of VLDL by the female rats under an obesogenic diet might directly affect the liver TAG concentration and its daily variation [48]. Only the females presented daily differences in the concentration of TAGs in the liver under both diets with lower levels at ZT15 than at ZT3 in both diets. Based on the results of this study, the ability of female rats to maintain the rhythmicity of VLDL packaging through PC synthesis may be essential to maintain the rhythmicity of TAG in the livers of female rats fed a cafeteria diet.

Since rats present nocturnal feeding, it is expected that the major intake of TAG from the diet occurs during the dark period [49]. The appearance of daily variations in the metabolites related to VLDL suggests their synthesis in a circadian manner. Thus, we propose that the female rats start to increase the VLDL level at ZT15 through the metabolites involved in the CDP-choline and PEMT methylation pathways to respond to the excess TAG derived from the obesogenic diet, increasing their secretion packaged in VLDL during the dark period.

In blood, previous studies have shown a positive correlation between VLDL and plasma TAG [50]. Our results have shown a negative correlation between both the liver PC and the plasma TAG in CAF-fed female animals. The lowest PC concentration was found at ZT3; consequently, the highest plasma TAG levels should be at the same time. This finding suggested that PC was used during the dark period to package the TAG derived from the diet in VLDL, which were finally secreted into the blood.

In summary, our results demonstrated that the metabolism related to the synthesis and packaging of VLDL in the liver changes in a daily manner, and these variations are affected by gender and diet. Notably, gender emerges as an essential factor in the capacity of the liver to manage a cafeteria diet, with females being more resistant to this diet. In this sense, female animals respond to the increased lipids derived from a cafeteria diet through the improvement of the daily variations in most of the metabolites related to the synthesis of VLDL. This could allow an increase in TAG exit from the liver during the dark period, reducing the possibility of suffering fatty liver disease. All these findings highlight the need to use both genders in nutritional interventions because the existence of different rhythmicity in the hepatic metabolism in each gender could significantly condition the effect of a specific diet. Actually, these results should be also taken into consideration in human experiments, since it has been demonstrated that there are significant differences between habit that have a direct impact on the circadian rhythms, such as duration of sleep, earlier timing, or slow-wave sleep [51]. Moreover, differences have been observed in some physiological parameters, such as plasma melatonin or body temperature, which are strongly associated with the circadian rhythms [52]. Particularly, regarding our results, sex differences have been demonstrated on the lipid kinetics, including the secretion of VLDL [53].

## Supplementary Materials

The following are available online at https://www.mdpi.com/2072-6643/12/4/1085/s1, Table S1: Biometric parameters in both male and female Fischer 344 rats fed either standard or obesogenic diet for 9 weeks. Table S2: Concentration of representative aqueous liver metabolites analysed by Nuclear Magnetic Resonance in female and male Fisher 344 rats fed with a chow diet and scarified at two different times, ZT3 or ZT15, Table S3: Concentration of representative lipid liver metabolites analysed by Nuclear Magnetic Resonance in female and male Fisher 344 rats fed with a chow diet and scarified at two different times, ZT3 or ZT15, Table S4: Concentration of representative aqueous liver metabolites analysed by Nuclear Magnetic Resonance in female and male Fisher 344 rats fed with a cafeteria diet and scarified at two different times, ZT3 or ZT15, Table S5: Concentration of representative lipid liver metabolites analysed by Nuclear Magnetic Resonance in female and male Fisher 344 rats fed with a cafeteria diet and scarified at two different times, ZT3 or ZT15.

## Highlights

Circadian rhythms differ between male and female rats. Obesity disrupt circadian rhythms in a gender manner. Obese female rats improve circadian rhythm of metabolites related to the VLDL synthesis. Obese female rats enhance the lipid liver profiling respect to male.
