# Supplementary material for: The Disruption of Liver Metabolic Circadian Rhythms by a Cafeteria Diet Is Sex-Dependent in Fischer 344 Rats
Source: Nutrients. 2020 Apr 14;12(4):1085. doi: 10.3390/nu12041085 (PMC7230270; doi:10.3390/nu12041085)
Supplement: Supplementary file 1 [file nutrients-12-01085-s001.pdf]

Supplementary Material

The disruption of liver metabolism circadian rhythms by an obesogenic diet is sex-dependent in Fischer 344 rats.

Author: Hector Palacios-Jordan

Corresponding: Miguel A. Rodríguez

Supplementary table 1. Biometric parameters in both male and female Fischer 344 rats fed either standard or obesogenic diet for 9 weeks.

	Female				Male			
	Standard		Cafeteria		Standard		Cafeteria	
	ZT3	ZT15	ZT3	ZT15	ZT3	ZT15	ZT3	ZT15
**Biometric parameters**								
Initial body weight (g)	151.35 ± 2.58	154.15 ± 2.39	153 ± 2.8	158.29 ± 2.19	228.48 ± 4.53	228.5 ± 9.63	207.46 ± 11.24	226.58 ± 2.45
Final body weight (g)	201.25 ± 4.72[a]	203.75 ± 4.27[a]	262.81 ± 5.584[b]	260.69 ± 7.57[b]	354.81 ± 6.54[a]	349.63 ± 15.24[a]	418.5 ± 6.78[b]	410.75 ± 4.68[b]
Caloric intake (KJ)	158.06 ± 2.52[a]	158.06 ± 2.42[a]	511.99 ± 39.11[b]	449.34 ± 7.39[b]	253.75 ± 4.04[a]	253.5 ± 7.48[a]	664.20 ± 14.86[b]	630.89 ± 23.15[b]

Female and male F344 rats were fed either a standard or obesogenic diet during 9 weeks. Data are expressed as the mean ± SEM (n = 8). One-way ANOVA and Duncan's post hoc tests were performed to compare differences between groups. Significant differences are represented by different letters (a, b). P-value < 0.05 were considered statistically significant.

Supplementary Table 2. Concentration of representative aqueous liver metabolites analysed by Nuclear Magnetic Resonance in female and male Fisher 344 rats fed with a chow diet and sacrificed at two different times, ZT3 or ZT15.

	Female		Male	
**Aqueous metabolites concentration (AU)**	**ZT3**	**ZT15**	**ZT3**	**ZT15**
3-Hydroxybutyrate	0.16 ± 0.01[a]	0.2 ± 0.03[a]	0.22 ± 0.01[a]	0.32 ± 0.05[b]
Formate	0.04 ± 0.003	0.05 ± 0.003	0.05 ± 0.003	0.05 ± 0.003
Fumarate	0.08 ± 0.01[a]	0.05 ± 0.004[bc]	0.06 ± 0.003[b]	0.04 ± 0.004[c]
Glucose 6-Phosphate	15.68 ± 1.3[ab]	14.73 ± 1.3[b]	20.22 ± 0.8[c]	18.38 ± 1.2[ac]
Lactate	6.67 ± 0.7	6.81 ± 1.1	6.09 ± 0.7	5.80 ± 0.7
Pyruvate	0.04 ± 0.02	0.05 ± 0.02	0.07 ± 0.01	0.03 ± 0.01
Succinate	0.77 ± 0.1	0.77 ± 0.1	0.88 ± 0.1	0.98 ± 0.1
Acetate	0.23 ± 0.02[a]	0.26 ± 0.02[a]	0.74 ± 0.2[b]	0.87 ± 0.1[b]
Choline	0.08 ± 0.01[a]	0.20 ± 0.03[b]	0.07 ± 0.01[a]	0.18 ± 0.02[b]
Betaine	2.29 ± 0.2[a]	2.56 ± 0.3[a]	1.28 ± 0.1[b]	1.55 ± 0.1[b]
Creatine	0.25 ± 0.02	0.22 ± 0.01	0.21 ± 0.01	0.19 ± 0.01
Creatine Phosphate	0.05 ± 0.002	0.06 ± 0.01	0.04 ± 0.001	0.04 ± 0.003
Creatinine	0.02 ± 0.002[a]	0.03 ± 0.003[a]	0.03 ± 0.003[a]	0.04 ± 0.01[b]
NAD+/NADH	0.17 ± 0.01	0.16 ± 0.02	0.19 ± 0.03	0.22 ± 0.02
Niacinamide	0.20 ± 0.01	0.19 ± 0.01	0.21 ± 0.01	0.21 ± 0.01
Inosine	0.69 ± 0.1[a]	0.70 ± 0.1[a]	1.56 ± 0.1[b]	1.32 ± 0.2[c]
Alanine	2.64 ± 0.1[a]	2.24 ± 0.2[a]	3.40 ± 0.2[b]	2.52 ± 0.2[a]
Glutamate	0.96 ± 0.1	0.99 ± 0.1	1.31 ± 0.1	1.21 ± 0.1
Glutamine	3.30 ± 0.2	4.11 ± 0.4	3.36 ± 0.2	3.92 ± 0.3
Glycine	1.44 ± 0.1[a]	1.38 ± 0.1[a]	1.04 ± 0.1[b]	0.99 ± 0.1[b]
Histidine	0.04 ± 0.002	0.05 ± 0.003	0.05 ± 0.004	0.04 ± 0.003
Isoleucine	0.19 ± 0.01[a]	0.22 ± 0.01[b]	0.19 ± 0.01[ab]	0.22 ± 0.01[b]
Leucine	0.60 ± 0.02	0.70 ± 0.1	0.59 ± 0.02	0.68 ± 0.03
Lysine	0.44 ± 0.02[ab]	0.64 ± 0.1[c]	0.38 ± 0.01[a]	0.50 ± 0.03[b]
Phenylalanine	0.38 ± 0.02	0.40 ± 0.03	0.34 ± 0.02	0.38 ± 0.01
Tyrosine	0.13 ± 0.01	0.12 ± 0.01	0.15 ± 0.004	0.17 ± 0.01
Valine	0.28 ± 0.01	0.32 ± 0.02	0.30 ± 0.01	0.35 ± 0.02
Ascorbate	0.56 ± 0.02[a]	0.47 ± 0.04[b]	0.65 ± 0.02[c]	0.54 ± 0.03[ab]

Standard female and male Fischer 344 rats were sacrificed at ZT3 and ZT15 fed with a chow diet for 9 weeks. Data are expressed as the mean ± SEM (n=8). All the metabolites were obtained by performing a nuclear magnetic resonance (NMR) analysis. One-way ANOVA and Duncan's post hoc test were performed to compare the values between groups and significant differences were represented with letters (a, b, c). P-value < 0.05 were considered statistically significant.

Supplementary Table 3. Concentration of representative lipid liver metabolites analysed by Nuclear Magnetic Resonance in female and male Fisher 344 rats fed with a chow diet and sacrificed at two different times, ZT3 or ZT15.

	Female		Male	
**Metabolites concentration (AU)**	**ZT3**	**ZT15**	**ZT3**	**ZT15**
Total cholesterol	3.73 ± 0.1	3.78 ± 0.1	3.55 ± 0.1	3.86 ± 0.2
Free cholesterol	3.58 ± 0.1	3.65 ± 0.1	3.40 ± 0.1	3.44 ± 0.2
Esterified cholesterol	1.49 ± 0.1	1.55 ± 0.1	0.26 ± 0.01	0.30 ± 0.1
Triglycerides	1.91 ± 0.2[a]	1.03 ± 0.1[b]	4.92 ± 1.4[c]	5.456 ± 2.1[c]
Diglycerides	2.68 ± 0.1[a]	2.36 ± 0.1[b]	0.81 ± 0.03[c]	0.80 ± 0.1[c]
Monoglycerides	1.12 ± 0.2	0.85 ± 0.1	0.65 ± 0.02	0.71 ± 0.04
Total phospholipids	15.15 ± 0.4[a]	16.15 ± 0.6[ab]	17.84 ± 0.2[bc]	18.32 ± 0.9[c]
Phosphatidylethanolamine	7.05 ± 0.2	7.46 ± 0.2	7.06 ± 0.1	7.94 ± 0.2
Phosphatidylserine	0.23 ± 0.01[a]	0.23 ± 0.01[a]	0.17 ± 0.01[b]	0.17 ± 0.01[b]
Phosphatidylcholine	14.54 ± 0.3[a]	15.32 ± 0.5[ab]	15.4 ± [ab]	16.3 ± 0.24[b]
Sphingomyelin	0.95 ± 0.02[a]	0.98 ± 0.04[a]	1.1 ± 0.03[a]	1.18 ± 0.1[b]
Plasmalogen	0.50 ± 0.03[a]	0.53 ± 0.03[a]	0.41 ± 0.02[b]	0.59 ± 0.1[a]
Omega-3	5.00 ± 0.1[a]	5.48 ± 0.2[b]	4.40 ± 0.1[c]	4.66 ± 0.3[bc]
ARA+EPA	8.26 ± 0.4[ab]	9.05 ± 0.4[b]	6.88 ± 0.2[c]	7.56 ± 0.6[ac]
Oleic acid	9.94 ± 0.7	8.08 ± 0.3	14.47 ± 2.2	14.61 ± 3.5
DHA	3.10 ± 0.1[a]	3.49 ± 0.2[b]	2.81 ± 0.1[a]	3.10 ± 0.3[a]
Linoleic acid	2.80 ± 0.2[a]	2.41 ± 0.2[a]	7.16 ± 0.8[b]	6.65 ± 0.5[b]

Standard female and male Fischer 344 rats were sacrificed at ZT3 and ZT15 fed with a chow diet for 9 weeks. Data are expressed as the mean ± SEM (n=8). All the metabolites were obtained by performing a nuclear magnetic resonance (NMR) analysis. One-way ANOVA and Duncan's post hoc test were performed to compare the values between groups and significant differences were represented with letters (a, b, c). P-value < 0.05 were considered statistically significant.

Supplementary Table 4. Concentration of representative aqueous liver metabolites analysed by Nuclear Magnetic Resonance in female and male Fisher 344 rats fed with a cafeteria diet and sacrificed at two different times, ZT3 or ZT15.

	Female		Male	
**Metabolites concentration (AU)**	**ZT3**	**ZT15**	**ZT3**	**ZT15**
3-Hydroxybutyrate	0.16 ± 0.01[a]	0.2 ± 0.03[a]	0.22 ± 0.01[a]	0.32 ± 0.05[b]
Formate	0.04 ± 0.003	0.05 ± 0.003	0.05 ± 0.003	0.05 ± 0.003
Fumarate	0.08 ± 0.01[a]	0.05 ± 0.004[bc]	0.06 ± 0.003[b]	0.04 ± 0.004[c]
Glucose 6-Phosphate	15.68 ± 1.3[ab]	14.73 ± 1.3[b]	20.22 ± 0.8[c]	18.38 ± 1.2[ac]
Lactate	6.67 ± 0.7	6.81 ± 1.1	6.09 ± 0.7	5.80 ± 0.7
Pyruvate	0.04 ± 0.02	0.05 ± 0.02	0.07 ± 0.01	0.03 ± 0.01
Succinate	0.77 ± 0.1	0.77 ± 0.1	0.88 ± 0.1	0.98 ± 0.1
Acetate	0.23 ± 0.02[a]	0.26 ± 0.02[a]	0.74 ± 0.2[b]	0.87 ± 0.1[b]
Choline	0.08 ± 0.01[a]	0.20 ± 0.03[b]	0.07 ± 0.01[a]	0.18 ± 0.02[b]
Betaine	2.29 ± 0.2[a]	2.56 ± 0.3[a]	1.28 ± 0.1[b]	1.55 ± 0.1[b]
Creatine	0.25 ± 0.02	0.22 ± 0.01	0.21 ± 0.01	0.19 ± 0.01
Creatine Phosphate	0.05 ± 0.002	0.06 ± 0.01	0.04 ± 0.001	0.04 ± 0.003
Creatinine	0.02 ± 0.002[a]	0.03 ± 0.003[a]	0.03 ± 0.003[a]	0.04 ± 0.01[b]
NAD+/NADH	0.17 ± 0.01	0.16 ± 0.02	0.19 ± 0.03	0.22 ± 0.02
Niacinamide	0.20 ± 0.01	0.19 ± 0.01	0.21 ± 0.01	0.21 ± 0.01
Inosine	0.69 ± 0.1[a]	0.70 ± 0.1[a]	1.56 ± 0.1[b]	1.32 ± 0.2[c]
Alanine	2.64 ± 0.1[a]	2.24 ± 0.2[a]	3.40 ± 0.2[b]	2.52 ± 0.2[a]
Glutamate	0.96 ± 0.1	0.99 ± 0.1	1.31 ± 0.1	1.21 ± 0.1
Glutamine	3.30 ± 0.2	4.11 ± 0.4	3.36 ± 0.2	3.92 ± 0.3
Glycine	1.44 ± 0.1[a]	1.38 ± 0.1[a]	1.04 ± 0.1[b]	0.99 ± 0.1[b]
Histidine	0.04 ± 0.002	0.05 ± 0.003	0.05 ± 0.004	0.04 ± 0.003
Isoleucine	0.19 ± 0.01[a]	0.22 ± 0.01[b]	0.19 ± 0.01[ab]	0.22 ± 0.01[b]
Leucine	0.60 ± 0.02	0.70 ± 0.1	0.59 ± 0.02	0.68 ± 0.03
Lysine	0.44 ± 0.02[ab]	0.64 ± 0.1[c]	0.38 ± 0.01[a]	0.50 ± 0.03[b]
Pehnylalanine	0.38 ± 0.02	0.40 ± 0.03	0.34 ± 0.02	0.38 ± 0.01
Tyrosine	0.13 ± 0.01	0.12 ± 0.01	0.15 ± 0.004	0.17 ± 0.01
Valine	0.28 ± 0.01	0.32 ± 0.02	0.30 ± 0.01	0.35 ± 0.02
Ascorbate	0.56 ± 0.02[a]	0.47 ± 0.04[b]	0.65 ± 0.02[c]	0.54 ± 0.03[ab]

Cafeteria female and male Fischer 344 rats were sacrificed at ZT3 and ZT15 fed with a chow diet for 9 weeks. Data are expressed as the mean ± SEM (n=8). All the metabolites were obtained by performing a nuclear magnetic resonance (NMR) analysis. One-way ANOVA and Duncan's post hoc test were performed to compare the values between groups and significant differences were represented with letters (a, b, c). P-value < 0.05 were considered statistically significant.

Supplementary Table 5. Concentration of representative lipid liver metabolites analysed by Nuclear Magnetic Resonance in female and male Fisher 344 rats fed with a cafeteria diet and sacrificed at two different times, ZT3 or ZT15.

	Female		Male	
**Metabolites concentration (AU)**	**ZT3**	**ZT15**	**ZT3**	**ZT15**
Total cholesterol	3.73 ± 0.1	3.78 ± 0.1	3.55 ± 0.1	3.86 ± 0.2
Free cholesterol	3.58 ± 0.1	3.65 ± 0.1	3.40 ± 0.1	3.44 ± 0.2
Esterified cholesterol	1.49 ± 0.1	1.55 ± 0.1	0.26 ± 0.01	0.30 ± 0.1
Tryglicerides	1.91 ± 0.2^{a}	1.03 ± 0.1^{b}	4.92 ± 1.4^{c}	5.456 ± 2.1^{c}
Diglycerides	2.68 ± 0.1^{a}	2.36 ± 0.1^{b}	0.81 ± 0.03^{c}	0.80 ± 0.1^{c}
Monoglycerides	1.12 ± 0.2	0.85 ± 0.1	0.65 ± 0.02	0.71 ± 0.04
Total phospholipids	15.15 ± 0.4^{a}	16.15 ± 0.6^{ab}	17.84 ± 0.2^{bc}	18.32 ± 0.9^{c}
Phosphatidylethanolamine	7.05 ± 0.2	7.46 ± 0.2	7.06 ± 0.1	7.94 ± 0.2
Phosphatidylserine	0.23 ± 0.01^{a}	0.23 ± 0.01^{a}	0.17 ± 0.01^{b}	0.17 ± 0.01^{b}
Phosphatidylcholine	14.54 ± 0.3^{a}	15.32 ± 0.5^{ab}	15.4 ± ^{ab}	16.3 ± 0.24^{b}
Sphingomyelin	0.95 ± 0.02^{a}	0.98 ± 0.04^{a}	1.1 ± 0.03^{a}	1.18 ± 0.1^{b}
Plasmalogen	0.50 ± 0.03^{a}	0.53 ± 0.03^{a}	0.41 ± 0.02^{b}	0.59 ± 0.1^{a}
Omega-3	5.00 ± 0.1^{a}	5.48 ± 0.2^{b}	4.40 ± 0.1^{c}	4.66 ± 0.3^{bc}
ARA+EPA	8.26 ± 0.4^{ab}	9.05 ± 0.4^{b}	6.88 ± 0.2^{c}	7.56 ± 0.6^{ac}
Oleic acid	9.94 ± 0.7	8.08 ± 0.3	14.47 ± 2.2	14.61 ± 3.5
DHA	3.10 ± 0.1^{a}	3.49 ± 0.2^{b}	2.81 ± 0.1^{a}	3.10 ± 0.3^{a}
Linoleic acid	2.80 ± 0.2^{a}	2.41 ± 0.2^{a}	7.16 ± 0.8^{b}	6.65 ± 0.5^{b}

Cafeteria female and male Fischer 344 rats were sacrificed at ZT3 and ZT15 fed with a chow diet for 9 weeks. Data are expressed as the mean ± SEM (n=8). All the metabolites were obtained by performing a nuclear magnetic resonance (NMR) analysis. One-way ANOVA and Duncan's post hoc test were performed to compare the values between groups and significant differences were represented with letters (a, b, c). P-value < 0.05 were considered statistically significant.
